# An assessment of patient-reported long-term outcomes following surgery for cauda equina syndrome

**DOI:** 10.1007/s00701-019-03973-7

**Published:** 2019-07-01

**Authors:** J. E. Hazelwood, I. Hoeritzauer, S. Pronin, A. K. Demetriades

**Affiliations:** 1 Centre for Clinical Brain Sciences, University of Edinburgh, Edinburgh, UK; 2 Department of Clinical Neurosciences, Western General Hospital, Telford Rd, Edinburgh, EH4 2XU UK; 3 Department of Neurosurgery, Western General Hospital, Crewe Road South, Edinburgh EH4 2XU, & New Royal Infirmary of Edinburgh 51 Little France Crescent, Old Dalkeith Road,, Edinburgh, EH16 4SA UK; 4 Edinburgh Spinal Surgery Outcome Studies Group, Edinburgh, UK

**Keywords:** Cauda equina syndrome, Long term, Outcome, Bladder dysfunction, Physical function, Pain

## Abstract

**Background:**

Data regarding long-term outcomes following surgery for cauda equina syndrome (CES) is scarce. In addition, these studies rely on patient descriptions of the presence or absence of symptoms, with no gradation of severity. This study aimed to assess long-term bladder, bowel, sexual and physical function using validated questionnaires in a CES cohort.

**Methods:**

A pre-existing ethically approved database was used to identify patients who had undergone surgery for CES between August 2013 and November 2014. Patients were contacted over a 1-month period between August and September 2017 and completed validated questionnaires via telephone, assessing bladder (Urinary Symptom Profile), bowel (Neurogenic Bowel Dysfunction Score), sexual dysfunction (Arizona Sexual Experiences Scale) and physical function (Physical Component Summary of SF-12 Questionnaire), with scores compared between those presenting with incomplete CES (CES-I) and CES with retention (CES-R). Patients were also asked which of their symptoms currently they would most value treatment for and what healthcare services they had accessed post-operatively.

**Results:**

Forty-six of 77 patients (response rate 72%, inclusion rate 60%) with a mean age of 45 years (21–83) and mean time since admission of 43 months (range 36–60) took part in the follow-up study. The prevalence of bladder dysfunction was 76%, bowel dysfunction 13%, sexual dysfunction 39% and physical dysfunction 48%. Patients presenting with CES-R had significantly worse long-term outcomes in bladder (stream domain), bowel and sexual function in compared to those with CES-I. Pain was chosen as the symptom patients would most value treatment for by 57%, but only 7% reported post-operative pain management referral.

**Conclusions:**

With a mean follow-up time of 43 months, these findings confirm the high prevalence of long-term bladder, sexual and physical dysfunction in CES patients and that a diagnosis of CES-R confers poorer outcomes. This study provides useful, objective data to guide the expectations of patients and clinicians.

## Introduction

Cauda equina syndrome (CES) is a neurosurgical and spinal orthopaedic emergency with potentially significant clinical and medicolegal consequences for both the patient and the medical team managing the condition. It is a relatively rare occurrence with an incidence of 0.3–1/100,000 in the general population and accounts for 2–6% of lumbar spine procedures [6]. However, it is difficult to establish the true incidence of the condition due to a lack of consensus on an exact definition of the syndrome and its sub-classes [5].

CES involves compression of the nerves of the cauda equina, most commonly caused by the herniation of an intervertebral disc [5]. This results in a constellation of symptoms related to a loss of cauda equina neural function including bladder, bowel and/or sexual dysfunction along with loss of saddle sensation, motor control or reflexes of the lower limbs [5]. The aim of surgical management is to restore normality of function by urgent decompression of the cauda equina nerve roots, but there is a risk that recovery may be only partial or absent entirely. It is these debilitating residual symptoms that contribute to the serious physical and socioeconomic consequences that can arise following CES.

The main body of research into CES has attempted to elucidate factors affecting post-operative outcomes, such as presentation characteristics and time to decompression [4, 7, 10, 13, 20]. Individual studies are largely equivocal, but meta-analyses conclude that earlier surgical decompression is beneficial for the patient, and that patients with urinary retention and overflow incontinence have poorer outcomes than those without [1, 3]. However, because the main area of investigation is prognostic factors *prior* to surgery, outcomes following surgery are understudied.

The few studies that do assess outcomes generally focus on mobility, pain or bladder function, with bowel or sexual function rarely investigated, possibly due to poor assessment and documentation [19]. Furthermore, they tend to be short term in design, with data collection limited to the first routine follow-up appointment, leading to a paucity of data regarding the long-term outcomes following CES surgery. For example, Srikandajarah et al. assess bladder outcome but only at around 3 months, with Korse et al. Investigating bladder, bowel and sexual function but only at 6 weeks [11, 23]. This is a short time into the patients’ recovery journey and it means that currently both clinicians and patients have little data on which to base long-term recovery expectations.

When long-term bladder function, bowel function and sexual function are measured, such as in Korse et al., studies rely on patient-reported data, with outcomes often not defined or measured using validated assessment tools [12, 24]. This dichotomises symptoms into functional or dysfunctional with no gradation of severity and it means that the data may not reflect the diverse range of residual symptoms which may be present.

### Aims

This study used validated questionnaires to objectively assess a range of long-term outcomes following CES surgery. The primary aim was to assess the patients’ current bladder, bowel and sexual function. Secondary aims assessed quality of life related to physical function, ability to return to work, what symptom patients would most value treatment for currently and long-term healthcare service use as reported by the patient.

## Materials and methods

### Participants and procedures

**Fig. 1:**
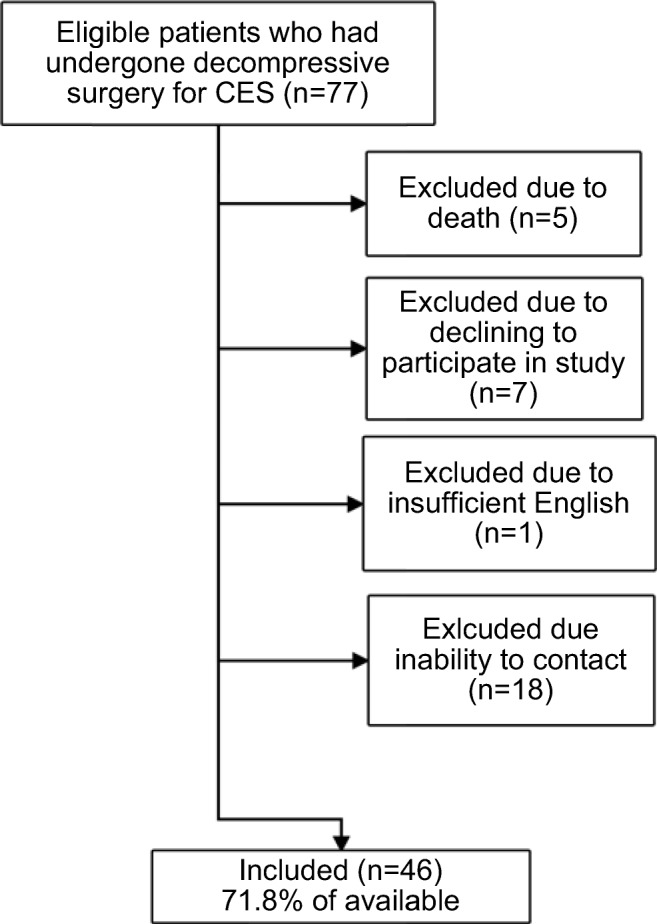
Patient inclusion

Seventy-seven patients who had attended the regional neurosurgical centre of the Western General Hospital, Edinburgh, between August 2013 and November 2014, were identified from a pre-existing ethically approved database of patients with suspected CES. This selected cohort had confirmed CES and relevant emergency treatment. After University of Edinburgh ethical review, the participants were contacted via telephone, gave informed consent and completed the questionnaire delivered by the author (JEH). Patients included were those with CES caused by degenerative disc disease; exclusion criteria comprised CES secondary to intradural or extradural tumours, inability to complete the questionnaire due to death, insufficient English or untraceable contact details (Fig. 1).

Following completion of the questionnaires, electronic records were used to confirm age and gender, and to assess whether the patient had incomplete CES (CES-I), with altered urinary sensation or loss of desire to void; or CES with retention (CES-R), with painless urinary retention and overflow incontinence [7].

### Questionnaire

The questionnaire contained two sections: validated and unvalidated. In the validated section, *bladder dysfunction* was assessed using the *Urinary Symptom Profile* (*USP*) with dysfunction defined by a score ≥ 1. This allows the breakdown of urinary symptoms into 3 domains of stress incontinence, overactive bladder (OAB) and low stream. Increasing scores indicate worsening dysfunction [8]. *Bowel dysfunction* was assessed using the *Neurogenic Bowel Dysfunction* (*NBD*) *Score*, which categorises bowel dysfunction into ‘very minor’ (score 0–6), ‘minor’ (7–9), ‘moderate’ (10–13) and ‘severe’ (14+), and rates overall bowel satisfaction out of 10 [14]. *Sexual dysfunction* was assessed using the *Arizona Sexual Experiences* (*ASEX*) *Scale*, where dysfunction is described by an overall score of ≥ 19, one domain ≥ 5 or 3 domains ≥ 4 [16]. Lastly, physical functioning was assessed using the Physical Component Summary (PCS) of the Short-Form 12 (SF-12) questionnaire with scores compared to the Scottish adult average data [26].

The unvalidated section was a semi-structured interview conducted by JEH. This assessed occupation status prior to CES; return to work following surgery; current status including any residual weakness/numbness/pain; whether pain prevents them from doing daily activities; use of any mobility aids; which of their symptoms they would most value relief from, and healthcare service use.

### Statistical analysis

Statistical analysis was performed using SPSS version 22 for Mac OS X (SPSS Inc., Chicago, IL). Independent *T* tests were used to analyse mean differences between the CES-I and CES-R, with statistical significance determined by a *p* value < 0.05.

## Results

Overall, 46/77 participants completed the study, generating a response rate of 72% and an inclusion rate of 60% (Fig. 1). The group comprised of 19 males and 27 females with a mean age of 45.4 years (range 21–83) and mean time since admission of 43.4 months (range 36–50). In total, 83% (*n* = 38) of participants had (CES-I) and 17% (*n* = 8) had CES-R. This proportion is similar to that of the initial cohort of 74 patients, with 81% CES-I and is therefore representative.

### Bladder function

On follow-up, 76% (*n* = 35) of patients suffered bladder dysfunction as defined by the USP (Table 1). Overactive bladder was the most frequently described symptom (72%), with stress incontinence (39%) and low stream (41%) affected at similar rates. The mean total USP score for all participants was 7.15 (± 7.17), with breakdown mean scores of overactive bladder 4.37 (± 4.72), low stream 1.59 (± 2.70) and stress incontinence 1.20 (± 2.07). Patients with CES-R demonstrated significantly more dysfunctional low stream scores (+ 2.77, *p* = 0.007), with no significant differences in the other USP domains.

**Table 1:** Patient characteristics at follow-up

Measure	*n* =	%	Mean score (±SD)
Urinary Symptoms Profile			
Overall Urinary Dysfunction Score	35	76	7.15 (± 7.17)
Stress incontinence	18	39	1.20 (± 2.07)
Overactive bladder	33	72	4.37 (± 4.72)
Low stream	19	41	1.59 (± 2.70)
Neurogenic Bowel Dysfunction Score			
Very minor	40	87	
Minor	4	9	
Moderate	0	0	
Severe	2	4	
Arizona Sexual Experiences Questionnaire			
Sexual dysfunction	18	39	
Physical function			
Working	29	63	
Working in a reduced capacity	6	13	
Not working	6	13	
Retired	5	11	

### Bowel function

On follow-up, 13% (*n* = 6) of participants reported bowel dysfunction with a severity of ‘minor’ or greater as defined by the NBDS. The mean score for satisfaction was 7.7/10, the median 9/10 and the mode 10/10. Bowel function was significantly worse in patients with CES-R, with a mean difference of + 4.13 compared to those with CES-I (*p* = 0.012).

### Sexual function

At follow-up 39% (*n* = 18) of patients reported sexual dysfunction as defined by the ASEX questionnaire. Patients were most commonly dysfunctional in the domains of sex drive (35% *n* = 16), ease (37% *n* = 17) and maintenance (35% *n* = 16) of arousal and ease of orgasm (39% *n* = 18) with orgasm satisfaction less affected (23% *n* = 11). Patients with CES-R had significantly worse sexual function, with a mean difference of + 6.76 (*p* = 0.009).

### Physical function and employment

**Fig. 2:**
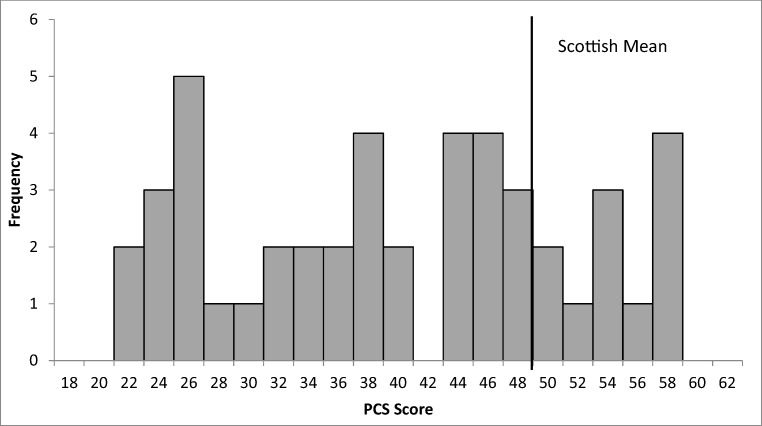
Histogram of patient SF-12 PCS distribution

The SF-12 demonstrated 48% (*n* = 22) of patients to have statistically significant poorer physical function than the Scottish adult average of 49 (±10.3) and the group mean PCS score of 39.2 (±11.3) to be markedly lower than this too (Fig. 2). Prior to admission 74% (*n* = 34) patients were in employment, with 15% (*n* = 7) unemployed and 11% (*n* = 5) retired. At follow-up, 71% (*n* = 29) of those of a working age were able to return to full employment, with 15% (*n* = 6) returning in a reduced capacity and 15% (*n* = 6) unable to work. The number of retired patients remained *n* = 5. There was no significant difference between CES-I and CES-R in PCS scores.

### Semi-structured interview

Residual symptoms were present in many with 70% (*n* = 32) reporting areas of sensory loss and 44% (*n* = 20) reporting current leg weakness, including 13% (*n* = 6) requiring walking aids to mobilise. Additionally, 70% (*n* = 32) of patients described themselves as suffering pain, of which the majority was back pain (35%, *n* = 16). This pain represents a significant barrier in 57% (*n* = 26) who state that pain prevents them from doing things in their daily lives.

**Fig. 3:**
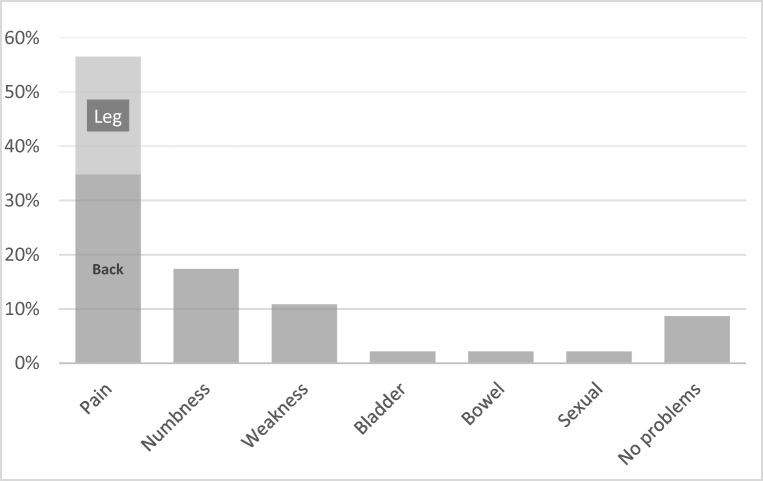
Patients’ symptom for which they would most value treatment

Despite a variety of residual symptoms, the majority of patients chose pain as the symptom that they would most value treatment for (57% *n* = 26). Back pain was highlighted as more important than leg pain with 35% (*n* = 16) stating it to be the symptom that they would most like to remove (Fig. 3).

### Review after discharge

**Fig. 4:**
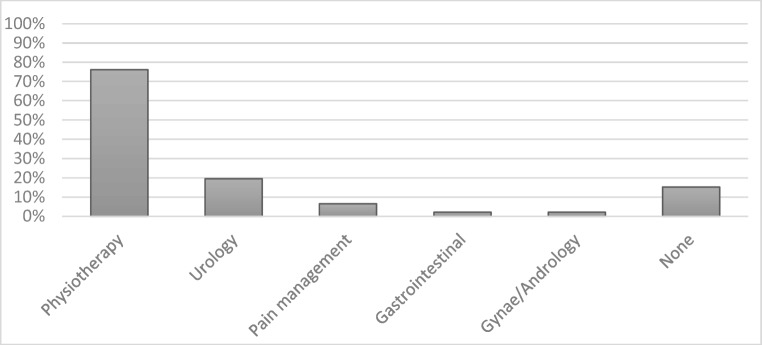
Patients’ reported post-discharge healthcare service use

Following hospital discharge, 85% (*n* = 39) of patients reported having contact with the healthcare service, the most common being community-based physiotherapy (76% *n* = 35). Fewer patients, 20% (*n* = 9), stated they had been referred to specialist urology services and fewer still 7% (*n* = 3) had been referred to the pain management team (Fig. 4).

## Discussion

This study aimed to assess the long-term outcomes following CES surgery by using validated questionnaires to quantify the symptoms currently experienced by a cohort of previous CES patients. Results demonstrated bladder, bowel and sexual dysfunction to be common problems within this population. Physical function was also shown to be significantly reduced in a large proportion of the patients, with many reporting persistent pain, sensory loss or weakness. Patients who had CES-R had significantly more stream-related bladder dysfunction, bowel dysfunction and sexual dysfunction, but no difference in physical functioning. Service use was assessed through semi-structured interview, with the majority of patients obtaining post-discharge physiotherapy, but few accessing urology or pain management services.

The main limitation of this study is the relatively small sample size (*n* = 46) and the risk of selection bias and social desirability bias when sourcing data from voluntarily responding patients. However, we achieved a follow-up rate of 71% using telephone interviews and attempted to minimise social desirability bias through a semi-structured interview approach. We feel our sample size to be satisfactory, since the median number of participants in CES studies is *n* = 14, and consider our inclusion rate to be adequate given the personal and intrusive nature of the questionnaires [24]. Whilst the USP is useful for determining the number of patients affected by bladder symptoms and in what way, it does not provide a scale to assess the impact of the symptoms on quality of life. As such, future studies could assess this through combining the USP with the recently validated SF-Qualiveen [12, 21]. Use of services post-discharge was patient reported. This was impossible to confirm due to patients coming from many regions with differing online record systems which may have impacted on the figures. However, urological and pain management interventions are often quite invasive or time intensive and the high rates of reported physiotherapy usage gives us confidence that these figures are unlikely to be grossly underestimated.

In highlighting the large burden of disease present in this patient population, our results broadly agree with previous literature in this area. However, the proportions of patients with residual symptoms differ in some categories.

Our study noted a much higher rate of bladder dysfunction (76%) than previous investigations, with McCarthy et al. finding 43% of patients to have bladder dysfunction at 5 years and Korse reporting 47% reducing to 41% on long-term follow-up [12, 15]. We hypothesise that this difference may be explained by the use of the objective USP score which is known to have a high sensitivity to a range of urological symptoms and patients would often report a symptom-free bladder, only to show dysfunction on the USP [27]. This conjecture is supported by the findings of Hellström et al. who describe how although only 41% of CES patients complained of bladder dysfunction, urodynamic findings were abnormal in 76% [9]. This potentially demonstrates an opportunity to improve symptoms in patients who are not aware of the possibility. The majority of bladder dysfunction was in the overactive bladder domain (72%), with less dysfunction related to stress incontinence or low stream. This is likely due to the neural damage sustained in CES that would preferentially affect detrusor innervation and function over pelvic floor strength or urethral patency [2].

Bowel dysfunction is the symptom with the greatest variation of reported prevalence in the literature and the results from this study continued this trend, describing a much lower rate than previously reported. Korse et al. describe a higher prevalence of 47% on initial follow-up, reducing to 43% over 13 years [12]. The rates reported by McCarthy et al. were higher still with 60% reporting ‘bowel disturbance’ as they were by Podnar; however, this study did not use validated questionnaires in data gathering [15, 18]. Using the neurogenic bowel dysfunction score allowed us to assess the degree of dysfunction present. Results showed that although the literature describes patients who complain of bowel disturbance following CES, few are affected to a quality-of-life-reducing level when investigated using validated methods. This is further supported by the high median and mode average in ‘bowel satisfaction’ with a lower mean value.

In regard to sexual function, our results demonstrated a lower prevalence of dysfunction (39%) compared to prior research, again likely caused by the method of outcome measurement. McCarthy et al. reported that 50% of patients had some degree of dysfunction, with Korse et al. finding dysfunction prevalence to be 56% at 2 months, marginally improving to 53% at 13 years [12, 15]. However, McCarthy et al. used different questionnaires to assess outcomes in males and females including the unvalidated Female Pelvic Floor Questionnaire. In Korse et al., the outcome was patient reported, not objectively assessed, and 11/19 were coded as ‘dysaesthesia of genital region’ or ‘not specified’. Whilst this may represent abnormal sexual function it does not necessarily imply dysfunction and may lead to an inflated prevalence.

Physical function is rarely objectively assessed in CES patients, but previous research agrees that the majority of patients score lower than the population average. McCarthy et al. assessed physical function using the Short-Form 36 questionnaire, a longer questionnaire from which the SF-12 was adapted and found CES patients to have significantly reduced function in the ‘Physical’ and ‘Role Physical’ domains [15].

Patients with CES-R demonstrated significantly poorer low stream bladder function, bowel function and sexual function in comparison to those with CES-I. This is likely due to the more serious nature of CES-R, which indicates compression and damage to nerves of the lumbo-sacral plexus and therefore more likely to result in subsequent permanent damage to the nerves supplying bladder, bowel and sexual function. Specifically, the low stream USP domain was worse in these patients due to some needing to self-catheterise as a result of loss of function due to CES. Few studies directly compare long-term outcomes between patients with CES-I and CES-R, with none to our knowledge assessing bladder, bowel, sexual and physical function. Gleave and Mcfarlane feel those with CES-R often have worse outcomes and this is supported by Kennedy et al. note that all 5 patients in a 19 patient study who had residual impairments at 2-year follow-up had urinary retention at presentation [7, 10]. However, McCarthy et al. found no significant differences between CES-I and CES-R in a range of outcomes [15]. A meta-analysis was performed, but was only able to report on urinary outcomes due to a lack of data present for other functions. This showed that patients with CES-R had relative risk of 2.58 of having bladder dysfunction compared to those with CES-I, although this result was not significant (95%CI 0.59–11.31) [3].

In a holistic approach, we also assessed function through the patients’ occupation status, symptom they would most value treatment for, and NHS service use post-discharge. In patients of working age, we found 71% were able to return to full employment, roughly matching the data from previous studies regarding spinal surgery which found 67% patients were able to return to work over a 3-month to 5-year follow-up [25].

Overall, 70% of patients declared that they suffered pain on follow-up, with 57% (*n* = 26) stating that it stops them from doing things in their daily lives. Furthermore, when asked to decide which symptom they would most value treatment for, the most commonly chosen option was pain, with back pain more important than leg pain. This is a considerably higher rate of pain than would be expected, given that the proportion of patients reporting leg or back pain 2 or more years after discectomy for radiculopathy is 17% [17]. Little literature has assessed the prevalence of pain as a long-term outcome, preferring to focus on other functions. However, a small study of 14 CES patients by Shapiro found that 28% suffered from chronic pain at 6-–60 month follow-up [22].

The patient-selected most important symptoms did not correlate with the patients’ stated use of NHS services. Despite 57% of this population describing their lives to be limited by pain and it being their chosen symptom for treatment, only 7% (*n* = 3) reported contact with the pain management team post-discharge suggesting that greater utilisation of this service could benefit this population.

Future studies should continue to follow this cohort and reassess for any future improvement in bladder, bowel, sexual or physical function using the questionnaires utilised by this study. Additional work could further investigate pain as an outcome in this population, using validated questionnaires.

## Conclusion

This long-term outcomes investigation of CES patients after surgery has identified continued abnormal bladder, bowel, sexual and physical dysfunction at a mean follow-up of 43 months.

Almost three quarters of patients continued to have bladder symptoms at long-term follow-up and almost 40% had sexual dysfunction. Bowel dysfunction was found to have less of an impact than previously suspected and physical dysfunction affected almost half of this cohort. Patients presenting with CES-R had significantly worse long-term outcomes in low stream bladder, bowel and sexual function in compared to those with CES-I.

Pain was identified as the symptom patients would most value treatment for, however, referrals for pain management did not correlate with the importance given to this symptom, highlighting the necessity of global assessment and management in this complex patient group.

We believe this to be the largest cohort of patients with CES investigated for long-term outcomes using validated questionnaires, and we hope this will provide some much needed data to guide the expectations of clinicians and patients throughout their CES diagnosis, operation and recovery process.

## Abbreviations

CES: Cauda equina syndrome
USP: Urinary Symptoms Profile
NBDS: Neurogenic Bowel Dysfunction Score
ASEX: Arizona Sexual Experiences Questionnaire
SF-12: Short-Form 12 Questionnaire
PCS: Physical Component Summary
